# Critical Examination of Müller Glia-Derived *in vivo* Neurogenesis in the Mouse Retina

**DOI:** 10.3389/fcell.2022.830382

**Published:** 2022-03-31

**Authors:** Ye Xie, Bo Chen

**Affiliations:** Departments of Ophthalmology and Neuroscience, Icahn School of Medicine at Mount Sinai, New York, NY, United States

**Keywords:** Müller glia, neurogenesis, mice, retina, fate mapping

## Abstract

Müller glia (MG) are a potential source of stem cells in the mammalian retina that could replenish lost retinal neurons for vision restoration. Unlike their counterpart in zebrafish, mammalian MG are quiescent and they do not spontaneously generate new retinal neurons. In recent years, extensive research efforts have been made to unlock the regenerative capabilities of Müller glia (MG) for *de novo* regeneration of retinal neurons in mice. Here, we discuss current research progress on MG-derived *in vivo* neurogenesis in the mouse retina, focusing on the use of stringent fate mapping techniques to evaluate and validate *de novo* regeneration of retinal neurons through the reprogramming of endogenous MG. Establishing stringent experimental criteria is critical for examining current and future studies on MG-derived regeneration of photoreceptors, retinal inter-neurons, and retinal ganglion cells.

## Introduction

The retina is a highly organized laminar structure composed of specialized nerve cells in the back of the eye. Loss of retinal neurons from either genetic deficiencies or environmental insults leads to vision impairment and blindness. The vast majority of vision loss results from the degeneration of two classes of retinal neurons, the light-sensing photoreceptors that mediate the first step in vision and retinal ganglion cells (RGCs), the output neurons in the retina that send visual information from the eye to the brain via their long axon projections. Degeneration of photoreceptors and RGCs is the leading cause of vision loss in major eye diseases, including age-related macular degeneration, diabetic retinopathy, and glaucoma. Therefore, photoreceptors and RGCs are in great need of being replenished.

Regenerative therapies hold great potential for vision restoration by replenishing lost retinal neurons. Significant efforts in developing retinal regenerative therapies involve transplantation of stem cell-derived photoreceptors into the host retina. The success of this approach requires the integration of newly transplanted photoreceptors into existing retinal circuitry. Although promising, stem cell-derived photoreceptor transplantation is not only technically challenging but initial integration of new photoreceptors was later (Pearson et al., 2012) shown to be at least partially a result of cytoplasmic material transfer from donor cells to endogenous photoreceptors of the host retina (Pearson et al., 2016; Santos-Ferreira et al., 2016; Singh et al., 2016; Waldron et al., 2018). An alternative approach is to unlock the retina’s own regenerative capability from MG (Too and Simunovic, 2021). In lower vertebrate species such as zebrafish, MG serve as ‘retinal stem cells’ (Raymond et al., 2006; Lenkowski and Raymond, 2014) and possess a remarkable capacity to regenerate all retinal neurons after damage and restore lost sight, establishing a powerful self-repair mechanism (Mensinger and Powers, 1999; Mensinger and Powers, 2007; Sherpa et al., 2008). MG are the primary glial cell type in the retina and their normal function is to provide structural, functional, and metabolic support for maintaining retinal homeostasis (Goldman, 2014). Although the homeostasis maintaining functions of zebrafish MG are similar to those of mammalian MG, zebrafish MG can spontaneously reenter the cell cycle in response to retinal damage and divide asymmetrically to produce a single progenitor-like cell. This multipotent daughter cell undergoes multiple rounds of cell division to generate a cluster of neurogenic progenitors, which then differentiate to produce all types of retinal neurons (Karl and Reh, 2010; Goldman, 2014; Wan and Goldman, 2016). Unlike their counterpart in lower vertebrates, mammalian MG barely reenter the cell cycle after retinal injuries. In the adult rat retina, only a small subset of MG can be stimulated to proliferate in response to excitotoxic injury, and newly produced neurons are very limited in numbers (Ooto et al., 2004). Similarly, neither NMDA-induced excitotoxic injury nor growth factor alone could induce substantial proliferation of MG in the mouse retina *in vivo*. When growth factors (EGF, FGF1, or a combination of FGF1 and insulin) were injected *in vivo* after NMDA-induced neuronal degeneration in the mouse retina, substantial MG proliferation occurs and some of the MG progeny differentiate into cells that express features of retinal interneurons such as amacrine cells (Karl et al., 2008). Interestingly, PNU-282987, an α7 nicotinic acetylcholine receptor (α7 nAChR) agonist, acts on the retinal pigment epithelium (RPE) to stimulate MG-derived neurogenesis in the adult mouse retina (Webster et al., 2019). During the past decade, many groups have sought to identify key pathways and transcription factors responsible for MG-mediated retinal repair in zebrafish and other species, with the hope of recapitulating the full regenerative processes in mammals. Having built on these findings, several recent high-profile *in vivo* studies show that after manipulating key regulators in the adult mouse retina, endogenous MG can be reprogrammed to generate multiple types of retinal neurons, including rod photoreceptors (Yao et al., 2018), bipolar or amacrine-like interneurons (Jorstad et al., 2017; Hoang et al., 2020), and RGCs (Zhou et al., 2020; Xiao et al., 2021).

In this Review, we discuss current research progress through *in vivo* studies on MG-derived neuronal regeneration in the adult mouse retina, focusing on stringent experimental methods and criteria, especially fate mapping techniques, which shall be used to evaluate and validate whether *de novo* regeneration of retinal neurons actually takes place from reprogrammed endogenous MG. And we will also describe prospects for *in vivo* regeneration research in the mouse retina using these stringent methods and criteria. We hope that this review will help establish stringent standards in MG-derived reprogramming research toward the goal of vision repair in the future.

### Research Progress on MG-Derived *in vivo* Neurogenesis in the Mouse Retina

In zebrafish, injury-induced re-expression of *Ascl1a* is required for MG dedifferentiation, proliferation, and retinal regeneration (Fausett et al., 2008; Ramachandran et al., 2010). However, *Ascl1* is not significantly upregulated after injury in the mouse retina, raising the hypothesis that the level of *Ascl1* is the key for unlocking the regenerative capabilities of MG in both species. Indeed, Reh et al. have shown that Ascl1 gene transfer, combined with NMDA-induced excitotoxic injury, is sufficient to activate the neurogenic potential of MG in juvenile mice (Ueki et al., 2015). They also found that during retinal development and maturation, the chromatin structure in mouse MG was in the “closed” state and Ascl1 accessibility decreased (Ueki et al., 2015; Vandenbosch et al., 2020). To unlock the regenerative capability of MG in older mice, Reh and colleagues combined *Ascl1* overexpression with application of TSA, a histone deacetylase inhibitor to increase the chromatin accessibility, and thus enabled a subset of MG to proliferate and generate retinal interneurons in the NMDA-injured adult mouse retina (Jorstad et al., 2017). Reh and colleagues further improved the efficiency of MG-derived regeneration of bipolar and amacrine-like retinal interneurons after a combined treatment of Ascl1, NMDA, and TSA (ANT) through suppression of STAT signaling or ablation of microglia (Jorstad et al., 2020; Todd et al., 2020). In their most recent study, Reh and colleagues demonstrate that Ascl1 in combination with the proneural transcription factor Atoh1 stimulated MG-derived neurogenesis in the absence of injury and largely drives an immature RGC-like cell fate (Todd et al., 2021).

MG-derived neurogenesis after retinal damage varies greatly among species from full regenerative capacity in zebrafish to partially capable regeneration in chicken and to very little spontaneous regeneration in mice (Karl and Reh, 2010). Through extensive studies using single-cell RNA sequencing (scRNA-seq), bulk RNA-seq, and assays for transposase-accessible chromatin with high-throughput sequencing (ATAC-seq), Hoang et al. identified changes in gene expression following NMDA and light-induced retinal damage in mouse, chicken, and zebrafish retinas. They also developed a computational tool, known as integrated regulatory network analysis (IReNA), to analyze gene expression profiles and chromatin accessibility to reconstruct MG regulatory networks in response to various stimuli. In their study, the Nuclear Factor I (NFI) family (NFIA/B/X) was identified as a key hub reverting reactive MG back to a quiescent state in the adult mouse retina after retinal injury. Conditional knockout of *Nfia/b/x* in the adult mouse retina led to reduced expression of resting MG-associated genes, as well as increased expression of cell-cycle-associated genes *Ccnd1* and *Ccnd3*. Importantly, the re-expression of the proneural gene *Ascl1*. Unlike those from the wild-type (WT) mouse retina, MG from the NFI-depleted mouse retina can be reactivated by NMDA-induced injury and a subset of them transdifferentiated into bipolar and amacrine-like interneurons (Hoang et al., 2020).

Being the most vulnerable two retinal cell types in major retinal degenerative diseases, photoreceptors or RGCs are the most desirable neuronal types to be replenished. Serving as an important signaling cascade in activating and maintaining the proliferation of neural stem cells, Wnt signaling and its effector β-catenin have been previously identified to play an essential role for injury-induced regenerative responses in the zebrafish retina (Wan et al., 2014). Interestingly, Wnt activation by inhibition of glycogen synthase kinase-3β (GSK-3β) was sufficient to reprogram MG to form MG-derived multipotent retinal progenitors that can differentiate into all types of retinal neurons in the absence of injury (Ramachandran et al., 2011). Inspired by the progress made in zebrafish, a previous study by Yao et al. showed that the mRNA levels of the Wnt pathway genes were upregulated after retinal injury and gene transfer of *β-catenin* was sufficient to stimulate MG to reenter the cell cycle and proliferate in the adult mouse retina in the absence of injury (Yao et al., 2016). Following one round of cell division after *β-catenin* gene transfer, MG can be further reprogrammed to generate new rod photoreceptors after a second gene transfer of three pro-rod transcription factors Otx2, Crx, and Nrl (Yao et al., 2018).

Very recently, two *in vivo* studies reported successful MG-to-RGC conversion with high efficiency in the adult mouse retina. The first study by Zhou et al. utilized the CRISPR-CasRx system to knock down the expression of *Ptbp1*, resulting in MG-to-RGC conversion in the adult mouse retina (Zhou et al., 2020). The second study by Xiao et al. reported highly efficient MG-to-RGC conversion after ectopic expression of Math5 and Brn3b, two transcription factors that promote the RGC fate (Xiao et al., 2021). Strikingly, these two studies also reported that newly generated RGC regrew their axons through long-distance travel within the optic nerve all the way to the target regions in the brain, re-establishing the retina-brain connection. Significantly, MG-derived regeneration of RGCs restored visual function in the NMDA-induced excitotoxicity model or a genetic model (*Brn3b* ^*AP/AP*^ knockin mutant mice) of RGC loss, respectively. Furthermore, both studies showed that a small proportion of reprogrammed MG was converted into amacrine cells.

### Evaluation Criteria for MG-Derived *in vivo* Neurogenesis in the Mouse Retina

With the hope of repairing damaged retinal circuitry in mammals, studies on MG-derived neurogenesis will continue to attract research enthusiasm for many years to come. However, we should caution against misinterpretations in the field of regenerative biology through *in vivo* cellular reprogramming (Breunig et al., 2007; Blackshaw and Sanes, 2021). Substantive concerns have been raised regarding *in vivo* glia-to-neuron conversion using AAV-mediated reprogramming techniques (Martin and Poche, 2019; Qian et al., 2021). In summary, the approaches used to validate the *de novo* regeneration of retinal neurons by reprogramming MG vary widely in these earlier studies (Table 1). Therefore, it is imperative to establish stringent evaluation criteria, focusing on fate mapping/lineage tracing techniques, in MG-derived regeneration research. In addition, we will discuss other supporting experimental methods that can be used to interpret MG-derived regeneration of retinal neurons, including single-cell RNA sequencing and morphological changes during cellular reprogramming.

**TABLE 1:** Summary of methods and criteria used to evaluate the MG-derived neurogenesis in adult mouse retina.

Treatment	Gene manipulation	Lineage tracing	Intermediate status capture	Suggested mechanism
NMDA injury + Ascl1 OE + TSA	Mouse genetics (*tetO-Ascl1-ires-GFP*)	Stringent genetic-based (*Glast-CreER;LNL-tTA or Rlbp1-CreER;LNL-tTA*)	Genetic-based scRNA-seq (FACS of Ascl1-GFP^+^ cells)	Transdifferentiation; Two-step reprogramming?

NMDA injury + *Nfi* knockout + GF	Mouse genetics (*Nfia/b/x^lox/lox^*)	Stringent genetic-based (*Glast-CreER;CAG-LSL-Sun1-GFP*)	Genetic-based scRNA-seq (FACS of Sun1-GFP^+^ cells)	Transdifferentiation; Two-step reprogramming?

No injury needed: 1, β-catenin OE 2, Otx2, Crx, Nrl	recombinant AAVs (GFAP-β-catenin) (GFAP-Otx2, Crx, Nrl)	Stringent genetic-based (*GFAP-Cre;Rosa26-tdTomato*)	Morphological visualization (AAV-GFAP-GFP and Rhodopsin-tdTomato)	Two-step reprogramming

No injury needed: *Ptbp1* downregulation	recombinant AAVs (GFAP-CasRx-*Ptbp1*)	Non-stringent genetic-based (AAV-Cre in *Ai9* reporter mice)	No	Transdifferentiation

No injury needed: Math5/Brn3b OE	recomninant AAVs (GFAP-Math5-Brn3b)	Non-stringent genetic-based (AAV-based GFP reporter in WT or *Glast-CreER* mice)	AAV-based scRNA-seq and morphological visualization (GFAP-Math5-Brn3b-GFP)	Transdifferentiation

OE: overexpression; GF: Growth factors.

### Using Stringent Fate Mapping Techniques to Lineage Trace MG in Reprogramming Research

Stringent fate mapping by genetic lineage tracing is the most reliable method to assess MG-derived neurogenesis. Indeed, a recent study used stringent genetic-based lineage tracing techniques to revisit astrocyte-to-neuron conversion in the mouse brain. Surprisingly, instead of bona fide conversion from reprogrammed glial cells, as reported in several high-profile studies, the “newly generated” neurons were found to have come from mislabeled endogenous neurons (Wang et al., 2021), emphasizing the importance of performing stringent fate mapping experiments universally in all *in vivo* reprogramming studies targeting glia-to-neuron conversion.

To examine the role of Ascl1 in MG-derived neurogenesis in the damaged mouse retina, Reh and colleagues performed stringent fate mapping experiments using two MG-specific tamoxifen-inducible Cre mouse lines, *Glast-CreER* and *Rlbp1-CreER*, to generate *Glast-CreER* (or *Rlbp1-CreER*)*;LNL-tTA;tetO-Ascl1-ires-GFP* mice. Ascl1-expressing MG were lineage traced with *GFP* expression (Ueki et al., 2015; Jorstad et al., 2017). In another stringently carried out study (Hoang et al., 2020), Hoang et al. profiled the transcriptomic and epigenetic changes of MG from injured retinas in *Glast-CreER;CAG-LSL-Sun1-GFP* mice, in which MG were lineage traced with Sun1-GFP expression labeling the inner nuclear membrane after tamoxifen induction. To further investigate the role of the *Nfi* gene (*Nfia/b/x*), which was down-regulated shortly after injury but elevated at later stages, Hoang et al. examined tamoxifen-induced *Glast-CreER;CAG-LSL-Sun1-GFP;Nfia/b/x* ^*lox/lox*^ mice, in which NFIs were selectively deleted in MG. A third MG-specific mouse line, *GFAP-Cre*, was used by Yao et al. in their two studies. To examine MG cell fate changes after AAV-mediated gene transfer of *β-catenin* and subsequent gene transfer of three pro-rod transcription factors (Otx2, Crx, and Nrl), Yao et al. generated *GFAP-Cre;Ai14 (Rosa26-tdTomato)* mice to genetically trace MG lineages independently of the AAV-mediated labeling system (Yao et al., 2016; Yao et al., 2018). Different from the two other Cre lines (*Glast-CreER* and *Rlbp1-CreER*), the *GFAP-Cre* line is not tamoxifen-inducible. Given the concern of the GFAP promoter driving transgene expression in neurons in the AAV-mediated expression system, selection of the *Glast-CreER* or *Rlbp1-CreER* line would be a better choice for fate mapping experiments in MG-derived neurogenesis studies.

However, stringent fate mapping experiments by genetically tracing the lineages of MG were not performed in two recent studies demonstrating a highly efficient conversion of MG to RGCs. In one study, MG were marked, in the presence or absence of *Ptbp1* downregulation, by injecting AAV-GFAP-GFP-Cre into the eyes of *Ai9 (Rosa-CAG-LSL-tdTomato-WPRE)* reporter mice (Zhou et al., 2020). As a result, MG were exclusively labeled by the AAV-mediated expression system. In another study, MG were marked in three different ways: Firstly by injecting AAV-GFAP-GFP (control) or AAV-GFAP-Math5-Brn3b-GFP into the wild-type mouse retina; Secondly by co-injecting AAV-GFAP-tdTomato-Cre with AAV-CAG-FLEX-GFP (control) or AAV-CAG-FLEX-Math5-Brn3b-GFP into the wild-type mouse retina; thirdly by injecting AAV-CAG-FLEX-GFP (control) or AAV-CAG-FLEX-Math5-Brn3b-GFP into the retinas of tamoxifen-induced *Glast-CreER* mice (Xiao et al., 2021). Consequently, all three MG labeling approaches exclusively relied on an AAV-mediated expression system. Collectively, neither of the studies used stringent fate mapping techniques to trace the lineages of MG after experimental manipulations of the reprogramming factors, leaving the possibility that some or all marked RGCs could have come from endogenous RGCs due to leaky expression of the AAV-mediated labeling system. Indeed, Wang et al. reported leaky expression of AAV-GFAP-Cre in endogenous neurons of the mouse brain, indicating that the AAV-mediated expression system is unsuitable for tracing glial cell lineages in reprogramming research to determine glia-to-neuron conversion. Moreover, Wang et al. showed that the cell type specificity of AAV-GFAP-mediated expression of a transgene in the mouse brain could be dramatically altered by the cargo it carries (Wang et al., 2021). Taken together, without performing stringent fate mapping experiments by genetically tracing the lineages of MG, a successful glia-to-neuron conversion could simply be a misinterpretation due to mislabeling of endogenous neurons, including MG-to-RGC conversion in the mouse retina.

### Using scRNA-Seq (Single-Cell RNA Sequencing) and Capturing the Morphological Changes During MG-Derived Neurogenesis

Besides stringent fate mapping techniques, visualizing the intermediate states of morphological changes in real-time is another strong proof of MG-derived neurogenesis. And *in vivo* time-lapse imaging would be the best way to visualize the morphological changes of MG when they undergo neuronal differentiation in the reprogramming research. However, *in vivo* time-lapse imaging to monitor the morphological changes of MG can be extremely technically challenging for practical use. Therefore, two other experimental methods can be useful in analyzing MG-derived neurogenesis, including scRNA sequencing and imaging morphological changes to capture progressive stages of MG-derived neuronal differentiation.

scRNA-Seq, a high-resolution gene expression analysis for profiling molecular features of cell populations, has been successfully used to reveal the identities of MG-derived cells after reprogramming manipulations. Reh and colleagues used fluorescence-activated cell sorting (FACS) to isolate lineage traced GFP^+^ MG from the *Glast-CreER;LNL-tTA;tetO-Ascl1-ires-GFP* mouse retina. scRNA-seq profiling of isolated GFP-labeled MG showed that ANT treatment reprogrammed MG into two groups of cells that were different from the original MG population. A larger cluster of reprogrammed MG showed transcriptomic profiling resembling retinal progenitor cells, while a relatively smaller cluster was composed of bipolar and amacrine-like interneurons (Jorstad et al., 2017). In their subsequent study, pseudo-time analysis of scRNA databases from different transitional time points showed the progression from MG to neuronal states after combined treatment of ANT with STAT inhibition (ANTSi) (Jorstad et al., 2020). Hoang et al. also performed scRNA-seq analysis to profile the identities of MG-derived cells. After receiving damage from NMDA and treatment with growth factors, lineage traced Sun1-GFP^+^ MG were isolated by FACS and analyzed by scRNA-seq profiling, and the results show that deletion of NFI can reprogram MG into two groups of cells that were different from the original MG population. Although a small cluster of reprogrammed MG transdifferentiated into bipolar and amacrine-like interneurons, the vast majority of MG were non-neurogenic with proliferative status (Hoang et al., 2020). Identification of interneuron-like cells and progenitor-like cells, which are normally not present in the mature mouse retina, is a clear indication of MG that undergoe identity changes during the reprogramming process. Although Xiao et al. also performed scRNA-seq to capture the intermediate states of MG-to-RGC transition (Xiao et al., 2021), they did not use stringent fate mapping techniques to isolate lineage traced MG. Therefore, genetic-based lineage tracing of MG, independent of the AAV-mediated labeling system, is a prerequisite for scRNA sequence analysis of cellular state changes of reprogrammed MG.

Visualization of cell morphological changes during the *in vivo* reprogramming process would identify the transitional cellular states of MG-derived neurogenesis. Reh and colleagues showed that the reprogrammed MGs were ‘hybrid’ cells with a morphological appearance similar to MG and bipolar neurons (Jorstad et al., 2017). Yao et al. performed AAV-GFAP-GFP and AAV-Rhodopsin-tdTomato co-injection to facilitate the visualization of *de novo* genesis of rod photoreceptors. Three characteristic morphological changes were captured in reprogrammed MG at progressive stages over time. At the initial stage, the Rhodopsin-tdTomato reporter was turned on in some reprogrammed MG indicating that these cells were undergoing rod differentiation. At the intermediate stage, reprogrammed MG divided asymmetrically into two daughter cells, with one daughter cell staying in the middle of the inner nuclear layer (INL) and the other daughter cell migrating to the outer nuclear layer (ONL) and growing rod outer/inner segments. At the terminal stage, the daughter cell that had migrated to ONL differentiated into a mature rod photoreceptor, while the other MG-derived daughter cell remained in the INL and eventually turned off the expression of the Rhodopsin-tdTomato reporter (Yao et al., 2018). However, we should be cautious about the cytoplasmic material transfer, which confounded the interpretation of donor-host photoreceptor transplantation (Boudreau-Pinsonneault and Cayouette, 2018). Therefore, showing the morphological changes in lineage traced MG would be a more stringent method to consider in the future. Zhou et al. used Ai9 reporter mice to reveal the progression of MG-to-RGC conversion. However, their results only showed a growing number of MG-derived RGCs, without providing authentic morphological changes during the process of MG-to-RGC conversion (Zhou et al., 2020). Xiao et al. generated a Brn3b-GFP reporter mouse line, in which GFP was simultaneously expressed with Brn3b to monitor the progress of MG-to-RGC conversion (Xiao et al., 2021). However, two prominent morphological changes essential for MG-to-RGC conversion were missing in these studies: first, the migration path of MG somas from the middle of INL crossing the inner plexiform layer before reaching the ganglion cell layer; second, the trajectory of axon growth path from the newly generated RGC somas going towards the optic nerve head to exit the retina followed by extension of the axons in the optic nerve before the RGC axons reach their brain targets.

### A Two-step Reprogramming or Direct MG-To-Neuron Transdifferentiation?

**FIGURE 1:**
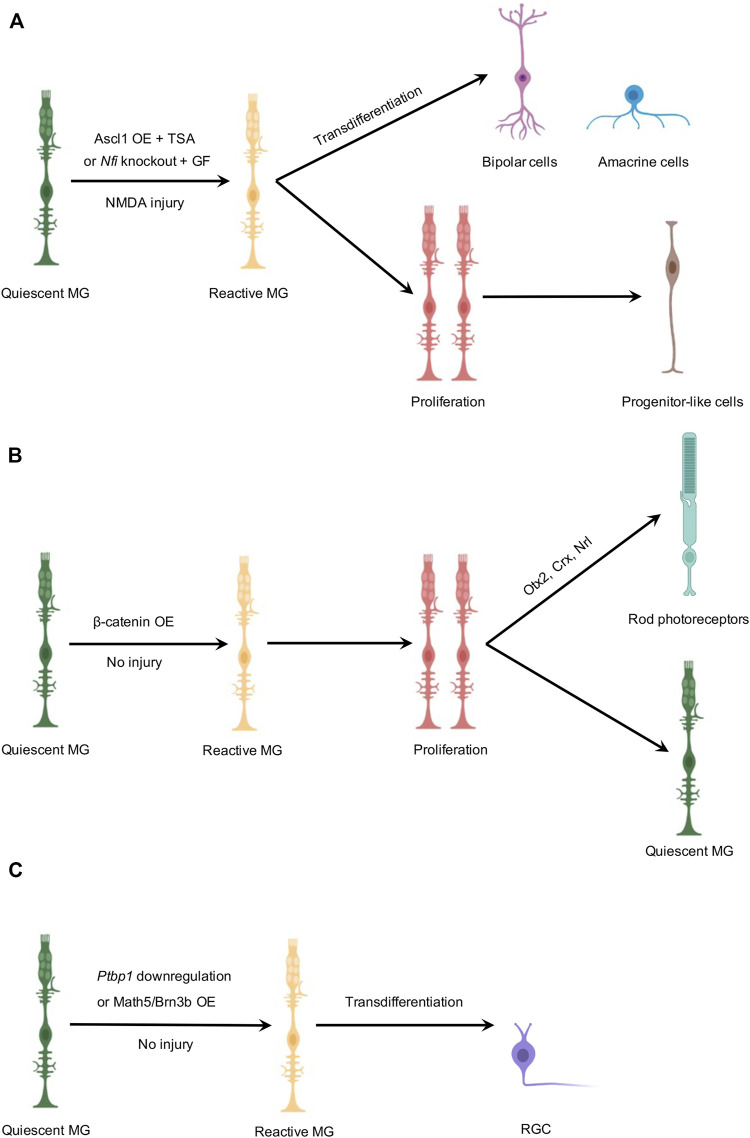
Schematic illustration of MG-derived *in vivo* neurogenesis in the adult mouse retina. **(A)** MG-derived regeneration of bipolar or amacrine-like cells. In the presence of NMDA-induced injury, Ascl1 overexpression combined with TSA or *Nfi* deletion combined with growth factors reprograms MG into two clusters of cells. One cell cluster is composed of bipolar and amacrine-like cells, and the other cell cluster contains progenitor-like cells that are mostly non-neurogenic. **(B)** MG-derived regeneration of rod photoreceptors. In the absence of retinal injury, β-catenin overexpression stimulates MG to reenter the cell cycle to proliferate as the first step. A subsequent gene transfer of Otx2/Crx/Nrl induces one daughter cell to differentiate into a rod photoreceptor, while the other daughter cell remains a quiescent MG. **(C)** MG-derived regeneration of RGCs. In the absence of retinal injury, *Ptbp1* downregulation or Math5/Brn3b overexpression converts MG into RGCs by direct transdifferentiation. However, genetic-based fate mapping experiments, independent of AAV-mediated gene transfer, were not performed in these studies to trace the lineage of MG after *Ptbp1* downregulation or Math5/Brn3b overexpression. OE, overexpression; GF, growth factors. Created with BioRender.com.

In zebrafish, MG first respond to retinal damage to transiently dedifferentiate into a stem cell state, reenter the cell cycle, undergo interkinetic nuclear migration, and an asymmetric division to generate a retinal progenitor. Next, this daughter cell proliferates to form a neurogenic cluster composed of multipotent retinal progenitor cells, which migrate along the radial fibers to the appropriate lamina, undergo neuronal differentiation, and eventually replenish lost neurons (Lenkowski and Raymond, 2014). Transdifferentiation, however, is a process in which a somatic cell switches its lineage to another differentiated cell type without undergoing an intermediate proliferative pluripotent stem cell state. Transdifferentiation can occur naturally and can also be induced experimentally. While MG-mediated retinal regeneration in zebrafish indicates a mechanism of two-step reprogramming, a recent study revealed that postmitotic bipolar cells can be re-specified to an amacrine cell fate in the zebrafish retina (Engerer et al., 2021), indicative of unanticipated plasticity of cell fate during retinal development. Thus, in studies of MG-derived *in vivo* neurogenesis in the mouse retina, a fundamental question to be addressed is whether it is a two-step reprogramming requiring MG to undergo proliferation before they differentiate into neurons or it is direct MG-to-neuron transdifferentiation/conversion (Figure 1). Reh and colleagues examined both possibilities in ANT-mediated neurogenesis by administering 5′-ethynyl-2′-deoxyuridine (EdU) daily to chemically trace MG that underwent proliferation. They found that some MG-derived cells that expressed the neuronal marker Otx2 also incorporated EdU. However, most MG-derived neurons were not labeled by EdU, suggesting that direct transdifferentiation could be the major mechanism (Jorstad et al., 2017). Hoang et al. also demonstrated that direct transdifferentiation could be the major mechanism in MG-derived neurogenesis of retinal interneurons since only 8.5% of Crx^+^ neurons and 6.1% of HuC/D; NeuN^+^ neurons were derived from *Nfi*-deleted MG that had incorporated EdU (Hoang et al., 2020). However, as consistent delivery and uptake of EdU could be technically challenging for practical use, it is difficult to guarantee that every proliferating MG incorporates EdU *in vivo* studies. The actual number of MG undergoing proliferation could have been heavily underestimated. A good practice to maximally label proliferating MG with EdU is to perform daily EdU injections covering the whole timeframe when MG are being treated. On the other hand, Yao et al. used a two-step reprogramming strategy to activate MG proliferation first before a subsequent gene transfer to induce rod differentiation (Yao et al., 2018), mimicking the natural process of MG-derived neurogenesis in zebrafish.

Interestingly, direct transdifferentiation/conversion was reported in two studies of MG-to-RGC conversion (Zhou et al., 2020; Xiao et al., 2021). Careful quantification of the total numbers of MG or RGC was necessary to confirm the results of direct cell conversion, as the number of MG loss was expected to be equivalent to the number gained from newly generated RGCs. As a therapeutic strategy for retinal repair, it is important to maintain the population of MG, as MG play a variety of essential roles to preserve retinal homeostasis, including maintaining retinal lamination (Vecino et al., 2016). Direct conversion of MG to RGCs and other retinal neurons would diminish the MG population, likely leading to retinal damage and degeneration.

### Mouse Genetic-Mediated *vs*. AAV-Mediated Gene Manipulation

Both mouse genetic manipulations and AAV-mediated gene manipulations are widely used for glia-derived *in vivo* neurogenesis studies. Here, we evaluate the strengths and weaknesses of both tools. The major strength of using genetic-based gene manipulation is that the manipulated gene(s) can be fairly uniformly overexpressed or conditionally knocked out when a Cre-responsive mouse line expressing the transgene(s) was crossed with a cell-type-specific Cre lineage tracing line. Two main limitations exist for using this tool for gene manipulations that include: 1) transgenic mouse lines are not always available for every gene, and crossing mouse lines could be time-consuming, especially when multiple genes need to be manipulated simultaneously. 2) Mosaic effects could occur when a Cre-responsive transgenic mouse line is crossed with a tamoxifen-induced Cre mouse line. Rueda et al. reported mosaic expression of the *Yap5SA* transgene in mouse MG when crossing with the *Glast-CreER* line specific to MG induced by tamoxifen (Rueda et al., 2019). Mosaic expression due to inefficiencies in Cre-mediated recombination might also occur when crossing with some *Rosa26* reporter mouse lines. Moreover, mosaic effects could be more profound when more than two mouse lines were crossed, evidenced by sparse MG labeling from *Glast-CreER;LNL-tTA;tetO-Ascl1-ires-GFP* mouse retina (Jorstad et al., 2017; Jorstad et al., 2020; Todd et al., 2020).

On the other hand, the major strengths of using AAV-mediated gene manipulation include: 1) manipulated genes are not restricted by the availability of mouse lines. 2) AAV vectors can be readily constructed for a wide range of experimental manipulations and purposes, and the preparation of recombinant AAVs is much less time-consuming. However, the weaknesses of using AAV-mediated gene manipulations also exist that include: 1) the limited packaging size of AAVs restricts its application to manipulate large genes. 2) the transduction efficiency, as well as the cell-type-specificity of AAV-mediated gene transfer, can be largely influenced by AAV serotypes (Klimczak et al., 2009). 3) Last but not least, the performance of promoter(s) for cell-type-specific expression could be influenced by the cargo it carries. The levels of leaky expression from glial cells to endogenous neurons vary greatly by individual genes driven by the same *GFAP* promoter (Wang et al., 2021). More recently, neuronal leaky expression driven by a well-known microglia-specific *CD68* promoter was also reported when lentivirus was used to target microglia *in vivo* (Rao et al., 2021), reemphasizing the necessity of using genetic-based stringent fate mapping techniques to evaluate *in vivo* cell reprogramming research.

## Conclusion

Performing stringent fate mapping experiments are extremely critical to evaluate *in vivo* reprogramming studies on MG-derived neurogenesis in the mouse retina. Having reviewed the methods and criteria used in recent *in vivo* MG-derived reprogramming studies in mice (Table 1) and discussed the strengths and weaknesses associated with each study, here we present a series of approaches that can be used to evaluate MG-derived neurogenesis studies: 1) First and foremost, stringent genetic-based fate mapping methods should be used across the board to trace the lineage of MG. A good practice is to use fate mapping mice generated by crossing MG-specific Cre lines, such as the *Glast-CreER* or *Rlbp1-CreER* line, with a *Rosa26* reporter line to trace the MG lineage. 2) Secondly, genetic-based scRNA-seq profiling and visualization of morphological changes will help reveal transitional cellular states when reprogrammed MG undergo neuronal differentiation. 3) Finally, EdU incorporation assays can be performed to examine whether MG-derived neurogenesis is resultant from direct transdifferentiation/conversion of MG in the absence of cell proliferation or from MG-derived progenitors that require MG proliferation before they undergo neuronal differentiation.

It is noteworthy that AAV-mediated gene transfer is a powerful tool in reprogramming research. However, AAV-based systems cannot be used exclusively to examine MG-derived neurogenesis due to possible leaky expression in endogenous retinal neurons. With a powerful gene manipulation tool at hand and stringent evaluation criteria to critically examine MG-derived *in vivo* neurogenesis in the mouse retina, we hope that unlocking the regenerative capacity of mammalian MG will continue to make strides in the important branch of regenerative medicine and visual neuroscience.
